# Unified Approach to the Motion Design for a Snake Robot Negotiating Complicated Pipe Structures

**DOI:** 10.3389/frobt.2021.629368

**Published:** 2021-05-03

**Authors:** Mariko Inazawa, Tatsuya Takemori, Motoyasu Tanaka, Fumitoshi Matsuno

**Affiliations:** ^1^Department of Mechanical Engineering and Science, Graduate School of Engineering, Kyoto University, Kyoto, Japan; ^2^Department of Mechanical and Intelligent Systems Engineering, The University of Electro-Communications, Tokyo, Japan

**Keywords:** snake robots, pipe inspection, bio-inspired robots, redundant robots, motion design

## Abstract

A unified method for designing the motion of a snake robot negotiating complicated pipe structures is presented. Such robots moving inside pipes must deal with various “obstacles,” such as junctions, bends, diameter changes, shears, and blockages. To surmount these obstacles, we propose a method that enables the robot to adapt to multiple pipe structures in a unified way. This method also applies to motion that is necessary to pass between the inside and the outside of a pipe. We designed the target form of the snake robot using two helices connected by an arbitrary shape. This method can be applied to various obstacles by designing a part of the target form specifically for given obstacles. The robot negotiates obstacles under shift control by employing a rolling motion. Considering the slip between the robot and the pipe, the model expands the method to cover cases where two helices have different properties. We demonstrated the effectiveness of the proposed method in various experiments.

## 1. Introduction

Despite their simple body configuration and lack of limbs, biological snakes move in a wide variety of environments, such as sandy and muddy places, in trees, and in narrow spaces. Inspired by biological snakes, snake robots with simple structures formed from repeating connecting modules have been developed and can perform various kinds of locomotion. They are expected to be used in dangerous situations, such as rescue work and infrastructure inspections, especially when spaces are narrow and inaccessible to humans, such as inside pipes. Controlling snake robots is a challenge because of their redundancy, and much research has been conducted to overcome this difficulty. The research that apply the motion observed in biological snakes, such as the undulation on the plane (Hirose, 1987) and the locomotion utilizing obstacles (Kano et al., 2018) into the engineering control of the snake robot has been done. Not only the motion but also the nervous system of biological snakes is utilized as the Central Pattern Generator (CPG) (Crespi and Ijspeert, 2008; Wu and Ma, 2013; Sartoretti et al., 2019).

Model-based control approach has also been studied. Several control methods have been developed that aid the convergence of control values toward reference values in modeling the interaction with snake robots and environments. These methods can be separated into two approaches. One considers the sideslip of the robot body (Saito et al., 2002; Mohammadi et al., 2015; Ariizumi et al., 2018), and the other considers non-holonomic constraints without sideslip (Matsuno and Sato, 2005; Tanaka et al., 2015; Nakajima et al., 2019). These methods have the advantages of simple environments and essentially planar surfaces but are unsuitable for complex or unknown environments because it is difficult to construct the dynamic model including the interaction with such environments.

To fulfill locomotion in such complicated environments for modeling, various designs of the whole form of the robot for effective locomotion have been proposed. These methods without modeling are beneficial in challenging environments, such as in narrow spaces and pipes in which the robot makes multiple points of contact along its length. Several gaits, e.g., *lateral rolling* and *pipe crawling*, have been realized by formulating a trajectory of joint angles as a gait function and changing several gait parameters that possess clear physical characteristics (Tesch et al., 2009; Rollinson and Choset, 2016). For complicated target forms for a snake robot, however, this approach is not feasible because it is difficult to formulate target joint angles directly.

To realize locomotion based on more complex target curves, methods of designing gaits by fitting a snake robot configuration to a target curve, which is designed as a continuous curve, have been proposed (Yamada and Hirose, 2006, 2008; Andersson, 2008; Hatton and Choset, 2010; Liljebäck et al., 2014). These methods make it possible to consider snake robot configurations as continuous curves, thus making it easy to design complicated forms. Takemori et al. (2018a) expanded Yamada's method (Yamada and Hirose, 2006) and proposed a method involving the design of a target curve by connecting simple shapes. They used their proposed method to design a target form that required the robot to partially lift the body around a flange on a pipe and achieved movement over the flange. Movements over rough terrain and climbing up ladders (Takemori et al., 2018b) were also accomplished.

**Figure 1:**
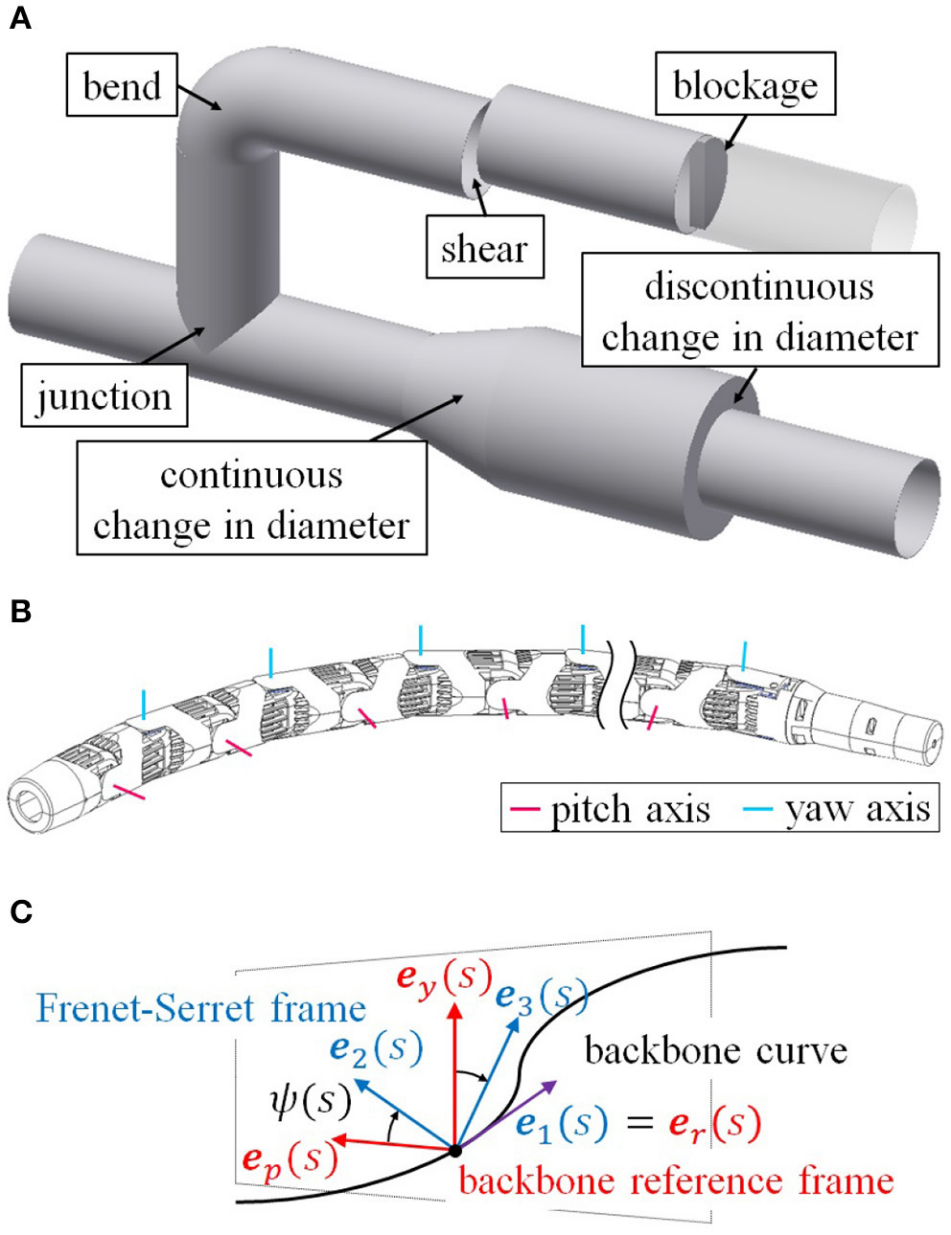
Concept of the research. **(A)** Various pipe structures. **(B)** Structure of a snake robot. **(C)** Definition of ψ(*s*).

The research of the snake robot moving inside a pipe is on the way to the final goal of our research that is to be able to perform pipe inspections with snake robots. As shown in Figure 1A, there are likely to be many “obstacles” that a robot moving inside pipes will have to navigate, including junctions, bends, continuous and discontinuous changes in pipe diameter, shears, and blockages. Some of these obstacles, such as bends, junctions, and continuous changes in pipe diameter are overcome with the previous methods (Kamegawa et al., 2011; Rollinson and Choset, 2016), whereas it has been difficult to deal with discontinuous diameter changes, shears, and blockages. Also, the robot is likely to encounter various kinds of obstacles one after another in an actual pipeline system. Since previous methods are designed only for an individual obstacle, many different methods are needed to deal with many kinds of obstacles serially. However, it is impractical to seamlessly switch between disparate control methods depending on the obstacle.

To get one step closer to the final goal, we address the negotiation of the complicated pipe structures in this paper. We propose a “unified” method that enables a snake robot to deal with all obstacles in Figure 1A, some of which have not yet been overcome and the others of which have already been overcome, just by altering the target form of the robot partially depending on the obstacle. Consequently, we can realize the motion control which serially negotiates various obstacles without switching to another method. In this method, the snake robot negotiates an obstacle by locally conforming to the shape of the obstacle while propelling itself forward through the pipe with a rolling motion. This method is improved by adjusting the rolling motion while considering the slip between the robot and a pipe, so as to make the method applicable to motion that includes two helices having different radii and pitches. Furthermore, this method creates a novel motion for entering and exiting a pipe. The entire motion of the robot is conducted remotely by an operator using simple inputs. We also demonstrate this unified motion, which is applicable to complicated pipe structures, to design target forms for junctions, bends, changes in pipe diameter, shears, blockages, and the inside-out motion as examples of its application. We also conduct experiments using this unified motion.

This research is based on Inazawa et al. (2020) and expands it by adding a model that considers the slip between pipe and robot in order to negotiate a change in diameter and to move from inside to outside a pipe. Finally, we carry out these experiments to verify the model.

## 2. Previous Work

This section introduces the previous work mentioned in section 1 in detail.

### 2.1. Approximation to a Continuous Curve

Methods for calculating the joint angles of the snake robot to approximate to a continuous curve were proposed in Yamada and Hirose (2006, 2008), Andersson (2008), Hatton and Choset (2010), and Liljebäck et al. (2014). Andersson (2008) proposed a method of fitting each joint to a target curve from head to tail for an articulated robot with universal joints. Hatton and Choset (2010) proposed *annealed chain fitting*, where approximation was conducted from a head by minimizing a cost function about the distance between each joint and a target curve. Liljebäck et al. (2014) proposed a method of fitting to a continuous curve generated by connecting points in three-dimensional space. Yamada and Hirose (2008) modeled a target curve for a snake robot (Yamada and Hirose, 2006) and proposed a method of obtaining a target angle for each joint by the curvature and torsion of the curve (Yamada and Hirose, 2008).

This Yamada's method can be applied to a robot with any joint configuration and is computationally inexpensive. Various gaits employing this method have been proposed (Kamegawa et al., 2009, 2011; Baba et al., 2010; Zhen et al., 2015; Zhou et al., 2017; Qi et al., 2018; Yaqub et al., 2019). Kamegawa et al. designed a helical target form and proposed *helical rolling motion* for moving on a pipe (Kamegawa et al., 2009; Baba et al., 2010). They also proposed a *helical wave propagation motion* (Qi et al., 2018) to negotiate a branch on the pipe. By sending a waveform down the helix, this motion enabled movement in the tangential direction of the helix, which a rolling motion cannot realize. Zhen et al. (2015) designed a curve superimposing a hump onto an arc and proposed a *rolling hump* that enables movement over obstacles using a rolling motion. Zhou et al. (2017) designed a target form including two helices and a connecting curve; by expanding and contracting like a spring, the robot achieved a movement outside a pipe with a changing diameter. Yaqub et al. (2019) designed a spiral curve having a gradually changing diameter, which enables the snake robot to move outside a pipe with a discontinuous change in diameter.

When a target curve becomes more complicated, it is difficult to express the spatial curve analytically. Also, the target joint angle cannot be calculated with Yamada's method (Yamada and Hirose, 2008) when torsion at a point diverges as the curvature is zero (Yamada and Hirose, 2006). To solve these problems, Takemori et al. (2018a) expanded Yamada's method (Yamada and Hirose, 2006) and proposed a method to design a target curve by connecting simple shapes, such as straight lines, circular arcs, and helices. This method enables an intuitive design of connecting shapes with familiar properties. Also, there is no need to calculate the curvature or torsion of a curve that is already known.

### 2.2. Motion Inside a Complicated Pipe

Rollinson and Choset (2016) proposed a method of compliance control in which the present form of the robot can be estimated from the joint angles using an extended Kalman filter based on gait parameters. This enabled semi-autonomous adaptation to a changing environment and locomotion inside pipes having bends, junctions, and continuous changes in diameter. This method is thought to be difficult to apply to great and discontinuous changes in diameter because the whole part of the robot winds around the pipe. Kamegawa et al. (2011) designed a target form by connecting a *bending helical curve* (Kamegawa et al., 2011) to a helix and realized movement inside a pipe with a bend. Some improvement is needed before these methods can be applied to shears and blockages, which require the robot to conform to obstacles elaborately.

## 3. Gait Design and Fitting Method

### 3.1. Shape Fitting Using a Backbone Curve

The snake robot in this study consists of alternating connected pitch-axis and yaw-axis joints, as shown in Figure 1B. The link length is *l*, and the relative angle of the *i*-th joint is θ_*i*_.

To start, we explain the approximation method with which we configure the snake robot to a target form (Yamada and Hirose, 2008). We begin with the representation of a spatial curve based on curvature and torsion. Let us consider the *Frenet–Serret frame*, which is an orthonormal basis (***e***_1_(*s*), ***e***_2_(*s*), ***e***_3_(*s*)) that depends on a single parameter *s* associated with the length along the curve. Moreover, ***e***_1_(*s*) is a vector tangential to the curve, ***e***_2_(*s*) is an inward vector normal to the curve, and ***e***_3_(*s*) is defined as ***e***_1_(*s*) × ***e***_2_(*s*). That is, the frame depends on the form of the curve. In addition, we need to consider the coordinate system that provides the orientation of the snake robot. We establish a *backbone reference frame* (***e***_*r*_(*s*),***e***_*p*_(*s*),***e***_*y*_(*s*)) on the curve. ***e***_*r*_(*s*) is the same vector as ***e***_1_(*s*), whereas ***e***_*p*_(*s*) and ***e***_*y*_(*s*) are vectors in the direction of the pitch-axis and yaw-axis, respectively.

As in Figure 1C, ψ(*s*) is defined as the twist angle between the Frenet–Serret frame and the backbone reference frame around ***e***_1_(*s*) and expressed by torsion τ(*s*) as

(1) $$\begin{array}{l} \psi \left(s\right)=\int_{0}^{s}\tau \left(\hat{s}\right)\text{d}\hat{s}+\psi_{0}, \end{array}$$

where ψ_0_ is an arbitrary constant of integration corresponding to the initial value of the twist angle. Changing ψ_0_ rotates the backbone reference frame around the curve and generates the *rolling motion*. The curvature around the pitch-axis and yaw-axis, denoted by κ_*p*_(*s*) and κ_*y*_(*s*), respectively, are expressible in terms of curvature κ(*s*) and ψ(*s*) as follows:

(2) $$\begin{array}{l} \kappa_{p}\left(s\right)=-\kappa \left(s\right)\sin\psi \left(s\right),\kappa_{y}\left(s\right)=\kappa \left(s\right)\cos\psi \left(s\right). \end{array}$$

Finally, we obtain the target angle of each joint as

(3) $$\begin{array}{l} \theta_{i}^{\text{d}}=\{\begin{array}{cc} \int_{s_{\text{h}}-\left(i+1\right)l}^{s_{\text{h}}-\left(i-1\right)l}\kappa_{p}\left(s\right)\text{d}s & \left(i:\text{odd}\right)\\ \int_{s_{\text{h}}-\left(i+1\right)l}^{s_{\text{h}}-\left(i-1\right)l}\kappa_{y}\left(s\right)\text{d}s & \left(i:\text{even}\right) \end{array}, \end{array}$$

where *s*_h_ is the head position of the snake robot on the target curve. The robot transforms itself smoothly under *shift control*, by which the change in *s*_h_ shifts the range corresponding to the robot's body within a target curve.

### 3.2. Backbone Curve Connecting Simple Shapes

Next, we explain the method of representing the target form as connected simple shapes for which the curvature and torsion are constant, such as straight lines, circular arcs, and helices (Takemori et al., 2018a). This method expands Yamada's method (Yamada and Hirose, 2008) to address the Frenet–Serret frames that are discontinuous at *connection-parts*, where simple shapes are brought together.

A connected simple shape is called a *segment*, and the *j*-th segment is referred to as *segment*-*j*(*j* ∈ ℤ). The connection-part between segment-*j* and segment-(*j* + 1) is referred to as *connection-part*-*j* at point *s* = *s*_*j*_. Points infinitesimally before and after the connection-part-*j* are denoted by *s*_*j*−_ and *s*_*j*+_, respectively. The curvature and torsion of segment-*j* are represented as κ_*j*_ and τ_*j*_, respectively. Using κ_*j*_ and τ_*j*_, the curvature κ(*s*) and torsion τ(*s*) of the target curve at *s*_*j*−1_ < *s* ≤ *s*_*j*_, which is equivalent to the point on segment-*j* within the connected segments, are defined as

(4) $$\begin{array}{l} \kappa \left(s\right)=\kappa_{j}\left(s-s_{j-1}\right),\;\tau \left(s\right)=\tau_{j}\left(s-s_{j-1}\right)\;\left(s_{j-1}<s\leq s_{j}\right). \end{array}$$

Let us next consider twists at the connection-part. The twist angle between ***e***_2_(*s*_*j*−_) and ***e***_2_(*s*_*j*+_) around ***e***_1_(*s*_*j*−_) is denoted by ${\hat{\psi }}_{j}$. To incorporate this twist angle into the calculation of shape fitting, (1) is replaced by

(5) $$\begin{array}{l} \psi \left(s\right)=\int_{0}^{s}\tau \left(\hat{s}\right)\text{d}\hat{s}+\psi_{0}+\underset{j}{\sum }{\hat{\psi }}_{j}u\left(s-s_{j}\right), \end{array}$$

where *u*(*s*) is the step function, for which its value is 0 if *s* < 0 and 1 if *s* ≥ 0.

In this study, we use straight lines, circular arcs, and helices as segments. For a straight line, the Frenet–Serret frame and the torsion cannot be determined; in this instance, we define the torsion as 0. An arc has a constant curvature and zero torsion and is defined by its radius *r*_*j*_ and central angle ϕ_*j*_. A helix has curvature and torsion that are both non-zero and constant and is defined by its radius *a*_*j*_, *b*_*j*_, and central angle ϕ_*j*_. Here, *b*_*j*_ = *p*_*j*_/2π is satisfied, where *p*_*j*_ is the pitch of the helix. Let us call the angle between the tangent of the helix and the plane perpendicular to the axis of the helix the *lead angle*, expressed as α = arctan(*p*_*j*_/2π*r*_*j*_). On the helix, ***e***_2_(*s*) is a vector directed vertically from the helix to the axis of the helix.

### 3.3. Shape Constraints

We consider the shape constraints for a target form resulting from the limits imposed on the joint angles. The maximum bending angle of a joint is represented as θ_max_. Whereas it is difficult to consider constraints in all states, here we only consider instances where the integration range in (3) includes separately only a circular arc and only a helix.

For the first instance, we let κ_c_ denote the curvature of the circular arc. From (3), the condition imposed to limit the target joint angle is given by

(6) $$\begin{array}{ll} \left|\theta_{i}^{\text{d}}\right| & =\{\begin{array}{ll} \left|\int_{s_{\text{h}}-(i+1)l}^{s_{\text{h}}-(i-1)l}-\kappa (s)\;\sin\psi (s)\text{d}s\right| & \left(i:\text{odd}\right)\\ \left|\int_{s_{\text{h}}-(i+1)l}^{s_{\text{h}}-(i-1)l}\kappa (s)\;\cos\;\psi (s)\text{d}s\right| & \left(i:\text{even}\right) \end{array}\\ \; & \leq \int_{-l}^{l}\kappa_{\text{c}}\text{d}s=2l\kappa_{\text{c}}\leq \theta_{\text{max}}. \end{array}$$

In the second instance, the curvature and torsion of the helix is denoted by κ_h_ and τ_h_, respectively. By substituting these into (5), ψ(*s*) is represented as

(7) $$\begin{array}{l} \psi \left(s\right)=\tau_{\text{h}}s+\psi_{0}. \end{array}$$

Substituting this into (2), the equation is represented as

(8) $$\begin{array}{l} \kappa_{p}\left(s\right)=-\kappa_{\text{h}}\sin\left(\tau_{\text{h}}s+\psi_{0}\right),\kappa_{y}\left(s\right)=\kappa_{\text{h}}\cos\left(\tau_{\text{h}}s+\psi_{0}\right). \end{array}$$

By substituting these into (3), the condition limiting the target joint angle is expressed as,

(9) $$\begin{array}{ll} \left|\theta_{i}^{\text{d}}\right| & =\{\begin{array}{ll} \left|\int_{s_{\text{h}}-(i+1)l}^{s_{\text{h}}-(i-1)l}-\kappa_{\text{h}}\;\sin\;(\tau_{\text{h}}s+\psi_{0})\text{d}s\right| & \left(i:\text{odd}\right)\\ \left|\int_{s_{\text{h}}-(i+1)l}^{s_{\text{h}}-(i-1)l}\kappa_{\text{h}}\;\cos\;(\tau_{\text{h}}s+\psi_{0})\text{d}s\right| & (i:\text{even})\; \end{array}\\ \; & \leq \max\left[\left|\int_{-l}^{l}\kappa_{\text{h}}\;\sin\;\left(\tau_{\text{h}}\bar{s}+x\right)\text{d}\bar{s}\right|(0\leq x<2\pi )\right]\;\\ \; & =\max\left[2\left|\frac{\kappa_{\text{h}}}{\tau_{\text{h}}}\;\sin\;(\tau_{\text{h}}l)\;\sin\;(x)\right|(0\leq x<2\pi )\right]\;\\ \; & \leq 2\left|\frac{\kappa_{\text{h}}}{\tau_{\text{h}}}\;\sin\;(\tau_{\text{h}}l)\right|\leq \theta_{\text{max}}, \end{array}$$

where

(10) $$\begin{array}{l} \bar{s}=s-\left(s_{\text{h}}-il\right), \end{array}$$

(11) $$\begin{array}{l} x=\tau_{\text{h}}\left(s_{\text{h}}-il\right)+\psi_{0}. \end{array}$$

## 4. Motion Design

**Figure 2:**
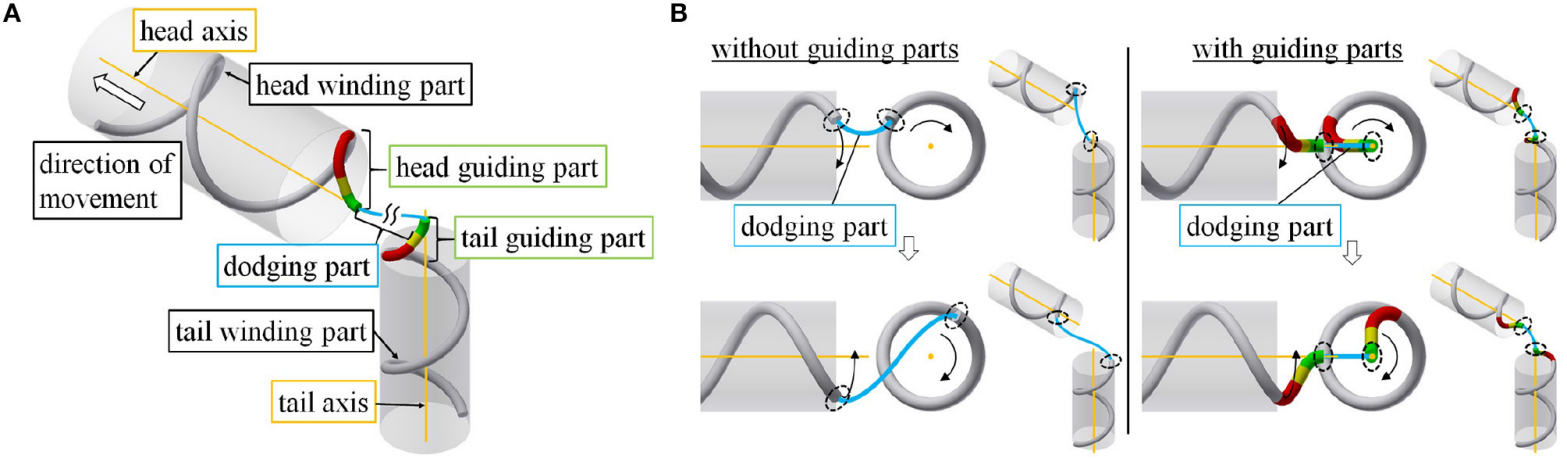
**(A)** Configuration of the target form for a snake robot negotiating a pipe. **(B)** Comparison between models with and without guiding parts. The black dotted ellipses represent the ends of the dodging part.

We now discuss the target form of the proposed motion. As shown in Figure 2A, the target form consists of two helices on straight pipes, called the *head winding part* near the head and the *tail winding part* near the tail; an axis for each winding part, called the *head axis* and *tail axis*; a guiding part from each winding part to its respective axis, called the *head guiding part* and *tail guiding part*; and a *dodging part*, which connects two guiding parts and dodges obstacles. Tailoring this dodging part to obstacles enables the robot to adapt to various obstacles. The robot negotiates an obstacle under shift control while moving its whole body from the tail winding part to the head winding part.

We mention the purpose of guiding each end of the dodging part from the winding part to its axis. First, consider an instance where each end of the dodging part (black dotted ellipse) is not on the axis of a pipe, as shown in the left panel of the Figure 2B. Although discussed in detail in section 4.2.1, here we remark that each winding part rotates around its axis according to commands from the shift control, and the relative position of the two end points of the dodging part changes. It is difficult to deform the dodging part (blue line) adequately in response to this change in the relative position of two end points. To solve this, we design guiding parts to guide each end of the dodging part (black dotted ellipse) onto each axis. Although each guiding part rotates around its axis, the relative position of the two ends of the dodging part no longer changes as shown in the right panel of Figure 2B. Therefore, the dodging part (blue line) easily adapts to an obstacle without deforming itself.

The winding and guiding parts are designed independently of an obstacle, whereas the dodging part is designed for it. In the following sections, we discuss the form design and the movement to realize the motion described above.

### 4.1. Form Design

We discuss the design of common parts regardless of obstacles. The parameters for these parts are shown in Table 1. We continue to describe each of these parts.

**Table 1:** Parameters describing segments that compose the form for pipe negotiation.

**Part**	**Seg no. j**	**Type**	**Parameter**	**${\hat{\psi }}_{j}$**
Tail winding part	1	Helix	(*a_j_, b_j_, ϕ_j_)* = (^t^*r*_w_, ^t^*p*_w_/2π, ^t^*β*_w_	0
Tail guiding part	2	Helix	(*a_j_, b_j_, ϕ_j_)* = (^t^*r*_in_, ^t^*p*_in_/2π, ^t^*β*_in_	0
	3	Straight line	$l_{j}{=}^{\text{t}}l_{\text{s}}$	$\frac{\pi }{2}$
	4	Circular arc	$\left(r_{j},\phi_{j}\right)=\left(r_{\text{c}},\frac{\pi }{2}{-}^{\text{t}}\alpha \right)$	$\phi_{\text{offset}}{+}^{\text{t}}\phi_{\text{rot}}$
Dodging part	–	–	–	–
Head guiding part	5 + *n*_d_	Circular arc	$\left(r_{j},\phi_{j}\right)=\left(r_{\text{c}},\frac{\pi }{2}{-}^{\text{h}}\alpha \right)$	0
	6 + *n*_d_	Straight line	$l_{j}{=}^{\text{h}}l_{\text{s}}$	$\frac{\pi }{2}$
	7 + *n*_d_	Helix	(*a_j_, b_j_, ϕ_j_)* = (^h^*r*_in_, ^h^*p*_in_/2π, ^h^*β*_in_	0
Head winding part	8 + *n*_d_	Helix	(*a_j_, b_j_, ϕ_j_)* = (^h^*r*_w_, ^h^*p*_w_/2π, ^h^*β*_w_	0

#### 4.1.1. Winding Part

The radius of the tail winding part ^t^*r*_w_ is given by (^t^*d*_pipe_/2) − *r*_link_, where ^t^*d*_pipe_ is the inner diameter of the pipe on the tail side and *r*_link_ is the link radius of the snake robot. The pitch of the tail winding part ^t^*p*_w_ is designed along with the tail guiding part, to which we turn next. The tail winding part is designed to be long enough to cover the whole body of the robot. Using the equation to obtain the length of the helix, the central angle of the tail winding part ^t^*β*_w_ is determined to satisfy

(12) lrobot≤l1=βtwrtw2+(ptw2π)2,

where *l*_robot_ is the total length of the snake robot. The radius, pitch, and central angle of the head winding part are denoted by ^h^*r*_w_, ^h^*p*_w_, and ^h^*β*_w_, respectively, and defined in a similar way to those of the tail winding part.

#### 4.1.2. Guiding Part

**Figure 3:**
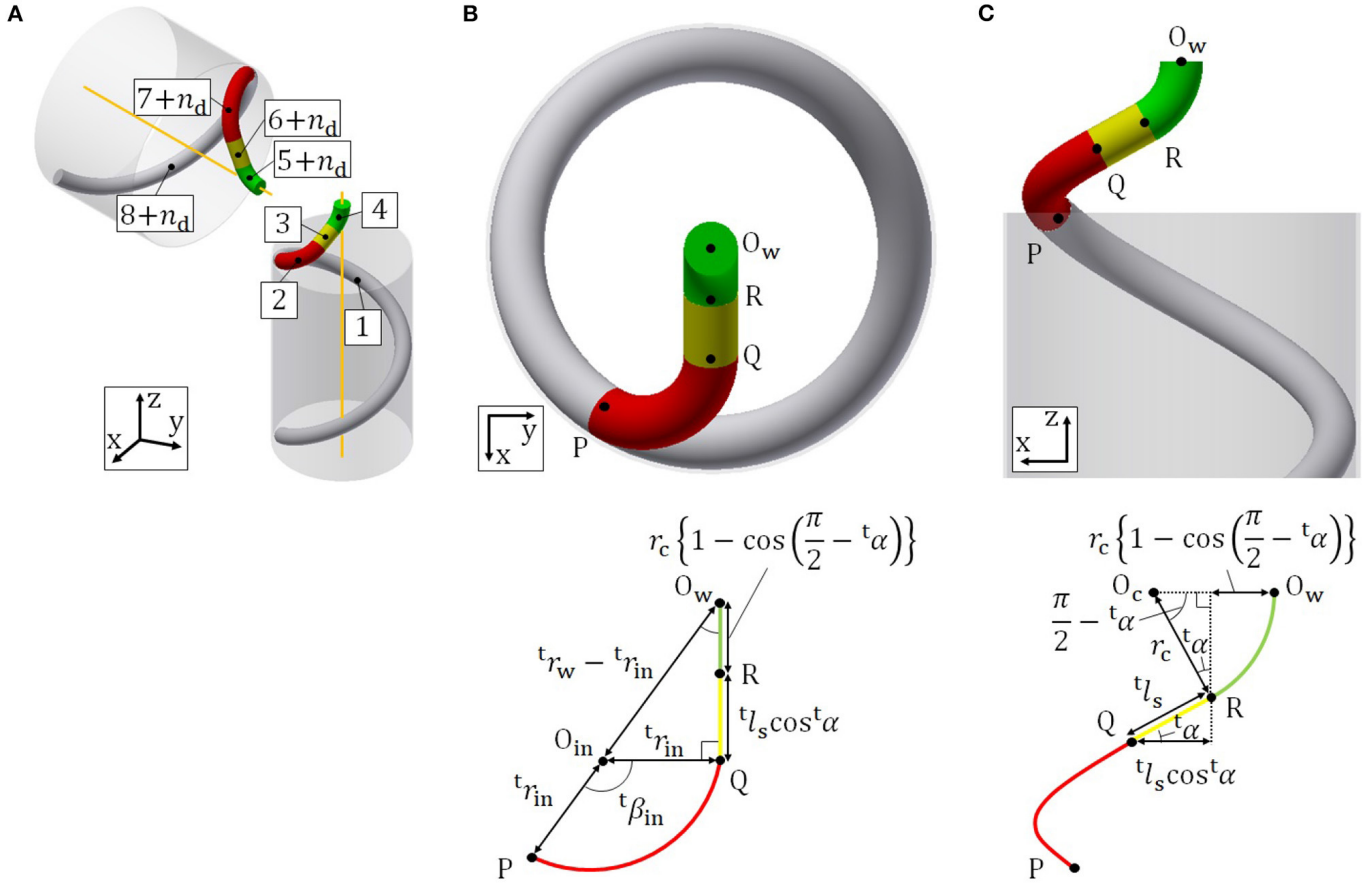
**(A)** Segment configuration of the guiding parts. **(B)** Schematic of the tail guiding part on the *xy* plane. **(C)** Schematic of the tail guiding part on the *xz* plane.

Figure 3A shows the segment configuration of the guiding parts. In Table 1, *n*_d_ is the number of segments comprising the dodging part. The head and tail guiding parts have similar shapes and parameters depending on the head and tail winding parts, respectively. For this reason, we treat only the tail guiding part, whose shape is determined by ^t^*p*_w_, the radius of segment-2 ^t^*r*_in_, and the radius of segment-4 *r*_c_.

A projection of the tail guiding part onto the *xy* plane in the absolute coordinate system *O* − *xyz*, where the *z* axis is equivalent to the axis of the pipe, is shown in Figure 3B. The points P, Q, R, O_w_ on the top of the figure are the connection-parts and have corresponding points on the bottom of the figure. O_w_ represents the center point of the gray circle in Figure 3B, which is the projection of segment-1 on the *xy* plane. Segment-2 is a helix, which changes the direction of the target curve toward the tail axis. The axis of segment-2 is designed to be parallel to the tail axis in order to simplify the geometrical calculation by turning all helices into a circle and an arc on the *xy* plane, as in Figure 3B. O_in_ represents the center point of the red circular arc in Figure 3B, which is the projection of segment-2 on the *xy* plane.

To realize this segment configuration, ^t^*p*_in_ and ^t^*β*_in_, and ^t^*l*_s_ are determined after the following calculation. Since segment-1 and segment-2 are connected continuously, the angle between the *xy* plane and the target curve at connection-part-1 is equal to the lead angle of segment-1. If the lead angle of segment-2 is equal to that of segment-1, the axis of segment-2 is perpendicular to the *xy* plane and parallel to that of segment-1. To ensure that the axes of segment-2 and the tail winding part are parallel to each other, the lead angle ^t^α must satisfy

(13) αt=arctanptw2πrtw=arctanptin2πrtin.

Then, we obtain ^t^*p*_in_ as

(14) ptin=rtinrtwptw.

A projection of the tail guiding part onto the *xz* plane is also shown in Figure 3C. The points P, Q, R, O_w_ on the top of the figure are the connection-parts and have corresponding points on the bottom of the figure. O_c_ represents the center point of the green circular arc in Figure 3C, which is the projection of segment-4 on the *xz* plane. ^t^*β*_in_ is derived by the geometric relationship shown in Figure 3B as

(15) βtin=∠OinQOw+∠OinOwQ=π2+arcsinrtinrtw−rtin.

To obtain ^t^*l*_s_, we firstly derive O_w_R and RQ in Figure 3B by the geometric relationship shown in Figure 3C as

(16) $$\begin{array}{ll} \text{RQ} & {=}^{\text{t}}l_{\text{s}}\cos^{\text{t}}\alpha , \end{array}$$

(17) $$\begin{array}{l} {\text{O}}_{\text{w}}\text{R}=r_{\text{c}}\left\{1-\cos\left(\frac{\pi }{2}{-}^{\text{t}}\alpha \right)\right\}. \end{array}$$

Then, ^t^*l*_s_ is given by (16), (17), and the geometric relationship shown in Figure 3B as

(18) lts=RQcostα=OwQ−OwRcostα =(rtw​−​rtin)2​−​rtin2−rc​{1−cos(π2​−​αt)}costα.

Note that ^t^*p*_w_ and ^t^*r*_in_ should be determined while satisfying ^t^*l*_s_ ≥ 0 for this segment configuration. In addition, the shape constraints described in section 3.3 also should be satisfied.

We introduce a parameter, ϕ_offset_, that is tuned by an operator and used to adjust the direction of the dodging part appropriately to the shape of the pipe.

#### 4.1.3. Dodging Part

The dodging part can be designed for a specific pipe structure. Section 5 provides examples relevant to a junction, bend, shear, blockage, and change in pipe diameter.

### 4.2. Procedure of Movement

**Figure 4:**
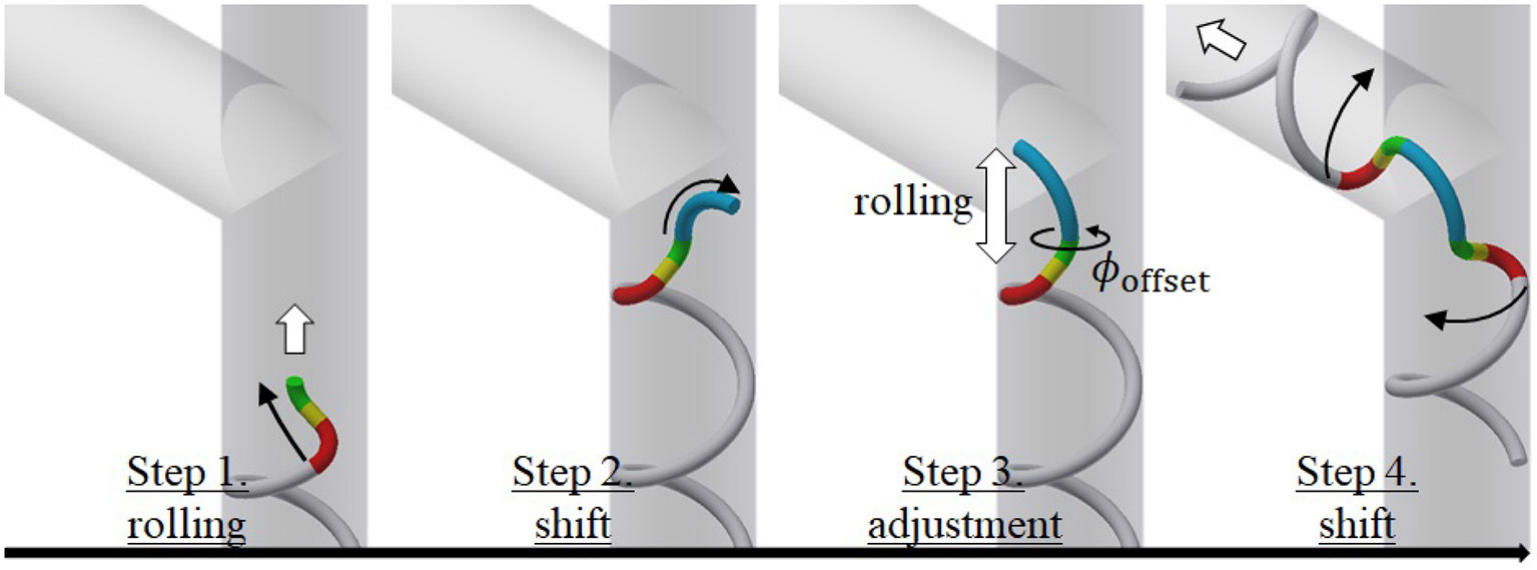
Procedure involved in movement.

We next explain the movement for the proposed motion. Figure 4 shows the procedural steps involved in negotiating an obstacle. Here, we use the target form for the junction presented in section 5. The four steps in Figure 4 are described as follows:

1. Step 1: Approach the obstacle using a rolling motion (rolling angle).
2. Step 2: Shift the head position to the dodging part under shift control (shift length).
3. Step 3: Adjust the position of the dodging part in the axial direction with a rolling motion and the direction using ϕ_offset_ (rolling angle and ϕ_offset_).
4. Step 4: Negotiate the obstacle under shift control (shift length).

Here, an operator commands the value in parentheses in each step. The operator can recognize the dodging part with Step 2 and adjust its position and direction with Step 3. The appropriate position and direction of the dodging part in Step 3 are different for each obstacle, as described in section 5.

#### 4.2.1. Fixing of Dodging Part

The position of the dodging part should be fixed to an environment while the robot is negotiating the pipe without colliding with the pipe in Step 4. The following figures illustrate instances for a junction. Here, no slip is assumed between the robot and pipe.

**Figure 5:**
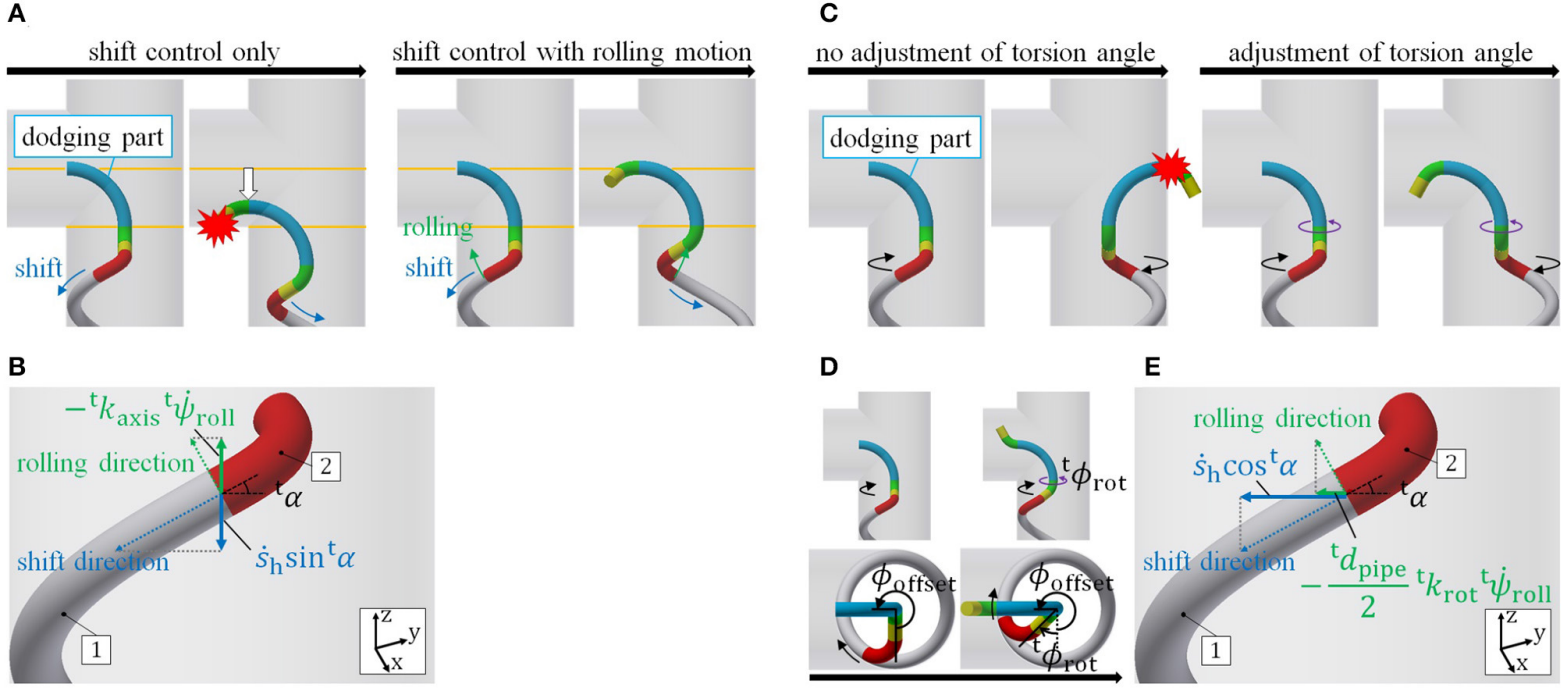
Fixing the dodging part. **(A)** Shift control executing rolling motion. **(B)** Displacement of the tail axis at connection-part-1. **(C)** Adjustment of the torsion angle. **(D)** Definition of ^t^*ϕ*_rot_. **(E)** Rotation around the tail axis at connection-part-1.

First, consider the displacement in the axial direction. As shown in the left panel of Figure 5A, just executing a shift control leads to the collapse of the target form because of the displacement of the dodging part in the axial direction. Therefore, this displacement caused by the shift control is canceled with a rolling motion, as shown in the right panel of Figure 5A. To this end, as shown in Figure 5B, the rolling velocity at connection-part-1 ψ∙troll is determined by the velocity of the shift control ṡ_h_,

(19) ψ˙troll=−s˙hsintαktaxis,

where ^t^*k*_axis_ is the displacement in the axial direction per unit rolling angle.

Second, consider the rotation around the axis. The rotation of the dodging part also breaks the target form because the direction of the dodging part becomes ill suited to the obstacle, as shown in Figure 5C. ^t^*ϕ*_rot_, the term in ${\hat{\psi }}_{4}$ in Table 1, is changed so as to cancel out the rotation of the dodging part (see Figure 5D). For this purpose, as shown in Figure 5E, ϕ∙trot, the time derivative of ^t^*ϕ*_rot_ is determined using ṡ_h_ and ψ∙troll by

(20) ϕ˙trot=−ktrotψ˙troll+2s˙hcostαdtpipe,

where ^t^*k*_rot_ is the rotation angle around the tail axis per unit rolling angle. ^t^*k*_axis_ and ^t^*k*_rot_ depend on the parameters of the tail winding part. It is difficult to analytically derive these values because of the fitting error of the robot and the slippage between robot and pipe. Therefore, these are actually measured in experiments in which only the rolling motion is performed.

#### 4.2.2. Derivation for Fixing the Dodging Part

We derive the appropriate value of ${\overset{∙}{\psi }}_{0}$, the time derivative of the initial twist angle ψ_0_, to realize ψ∙troll and ψ∙hroll in the previous section.

The point *a* away from the head of the robot is *s*_r_ = *s*_h_ − *a* (0 ≤ *a* ≤ *l*_robot_) on the target curve. From (5), $\overset{∙}{\psi }\left(s_{\text{r}}\right)$, the rolling velocity at *s* = *s*_r_, is expressed as

(21) $$\begin{array}{l} \overset{∙}{\psi }\left(s_{\text{r}}\right)=\overset{∙}{s_{\text{r}}}\frac{\text{d}}{\text{d}s_{\text{r}}}\int_{0}^{s_{\text{r}}}\tau \left(\hat{s}\right)\text{d}\hat{s}+{\overset{∙}{\psi }}_{0}+\frac{\text{d}}{\text{d}t}\underset{j}{\sum }{\hat{\psi }}_{j}u\left(s_{\text{r}}-s_{j}\right). \end{array}$$

After substituting *s*_r_ = *s*_h_ − *a* and rearranging the equation, this equation is expressed as

(22) $$\begin{array}{r} \overset{∙}{\psi }\left(s_{\text{h}}-a\right)=\tau \left(s_{\text{h}}-a\right){\overset{∙}{s}}_{\text{h}}+{\overset{∙}{\psi }}_{0}+\sum_{j}{\overset{∙}{\hat{\psi }}}_{j}u\left(s_{\text{h}}-a-s_{j}\right)\\ +\sum_{j}{\hat{\psi }}_{j}\delta \left(s_{\text{h}}-a-s_{j}\right){\overset{∙}{s}}_{\text{h}}, \end{array}$$

where δ is the impulse function. The impulse function has zero value except at the connection-part, where *s*_h_ − *a* − *s*_*j*_ = 0. On the other hand, the impulse function has non-zero value at the connection-part. However, it doesn't have any effect on the overall movement of the robot because the length of the connection-part is zero. Therefore, the last term on the right-hand side is negligible. The points on the tail winding part and on the head winding part in the target curve are represented as $s_{\text{r}}=s_{\text{h}}{-}^{\text{t}}a$ and $s_{\text{r}}=s_{\text{h}}{-}^{\text{h}}a$, respectively. To realize $\overset{∙}{\psi }\left(s_{\text{h}}{-}^{\text{t}}a\right){=}^{\text{t}}{\overset{∙}{\psi }}_{\text{roll}}$ on the tail winding part and $\overset{∙}{\psi }\left(s_{\text{h}}{-}^{\text{h}}a\right){=}^{\text{h}}{\overset{∙}{\psi }}_{\text{roll}}$ on the head winding part, the desired values of ${\overset{∙}{\psi }}_{0}$ on the tail winding part and on the head winding part are given by

(23) ψ˙troll=ψ˙0+τtws˙h,

(24) ψ˙hroll=ψ˙0+τhws˙h+ϕ˙trot−ϕ˙hrot,

where ^t^*τ*_w_ and ^h^*τ*_w_ denote the torsions of the tail and head winding parts, respectively.

When both of these winding parts have the same radius, ψ∙troll=ϕ∙hroll, ^t^*τ*_w_ = ^h^*τ*_w_, and ϕ∙trot=ϕ∙hrot. Therefore, (23) and (24) are satisfied simultaneously. This means that the fixing of the dodging part can be realized rigidly without the slip between the robot and pipe, which depends on the frictional condition and model.

However, when both winding parts have different radii, ψ∙troll≠ψ∙hroll, ^t^*τ*_w_ ≠ ^h^*τ*_w_ and ϕ∙trot≠ϕ∙hrot. Therefore, there is no ${\overset{∙}{\psi }}_{0}$ that satisfies (23) and (24) at the same time. For any value of ${\overset{∙}{\psi }}_{0}$, the slip occurs between the robot and pipe on one or both of the head and tail winding parts. This slip is possible to prevent the fixing of the dodging part which is based on the assumption of no slip. Hence, the model including the slip between the robot and pipe is required to realize the fixing of the dodging part.

#### 4.2.3. Model Including Slip Between Robot and Pipe

We next consider a model that includes the slip between the robot and pipe, and aim to derive the relationship between the shift velocity, ${\overset{∙}{\psi }}_{0}$, and ${\overset{∙}{\phi }}_{\text{diff}}$ to fix the dodging part against the pipe, where

(25) $$\begin{array}{l} {\overset{∙}{\phi }}_{\text{diff}}{=}^{\text{t}}{\overset{∙}{\phi }}_{\text{rot}}{-}^{\text{h}}{\overset{∙}{\phi }}_{\text{rot}}+\omega_{\text{slip}}. \end{array}$$

Here, ω_slip_ is the sum of the slip angular velocity around the axis on the tail and head winding parts.

Let us assume viscous friction between robot and pipe as in Saito et al. (2002), Liljebäck et al. (2010), Ariizumi and Matsuno (2017), and Ariizumi et al. (2018). The friction is assumed to be proportional to the normal force from the pipe as in Hicks and Ito (2005) and Ariizumi and Matsuno (2017). The normal force is considered to work equally along the body of the robot and is represented as *T* = ρ*l*_cont_, which is proportional to the contact length *l*_cont_. ρ is the coefficient of pressure per unit contact length. Here, the equilibrium of force in the axial direction is represented as

(26) $$\begin{array}{l} \mu^{\text{t}}v_{\text{slip}}\rho^{\text{t}}l_{\text{w}}+\mu^{\text{h}}v_{\text{slip}}\rho^{\text{h}}l_{\text{w}}=0, \end{array}$$

where μ is the coefficient of friction, and ^t^*v*_slip_ and ^h^*v*_slip_ are the slip velocities in the axial direction on the tail and head winding parts, respectively. Also, ^t^*l*_w_ and ^h^*l*_w_ are respectively the lengths of the tail and head winding parts within the range corresponding to the robot's body in the target curve. We next consider the velocity of the dodging part against the pipe. In order to fix the dodging part against the pipe, we consider the velocity occurring by shift control, rolling motion, and slip, and make its value zero. Since the target form is connected continuously, the two ends of the dodging part have the same velocity against the pipe *v*_dod_. Our purpose is to make *v*_dod_ zero.

##### 4.2.3.1. Change in Pipe Diameter

**Figure 6:**
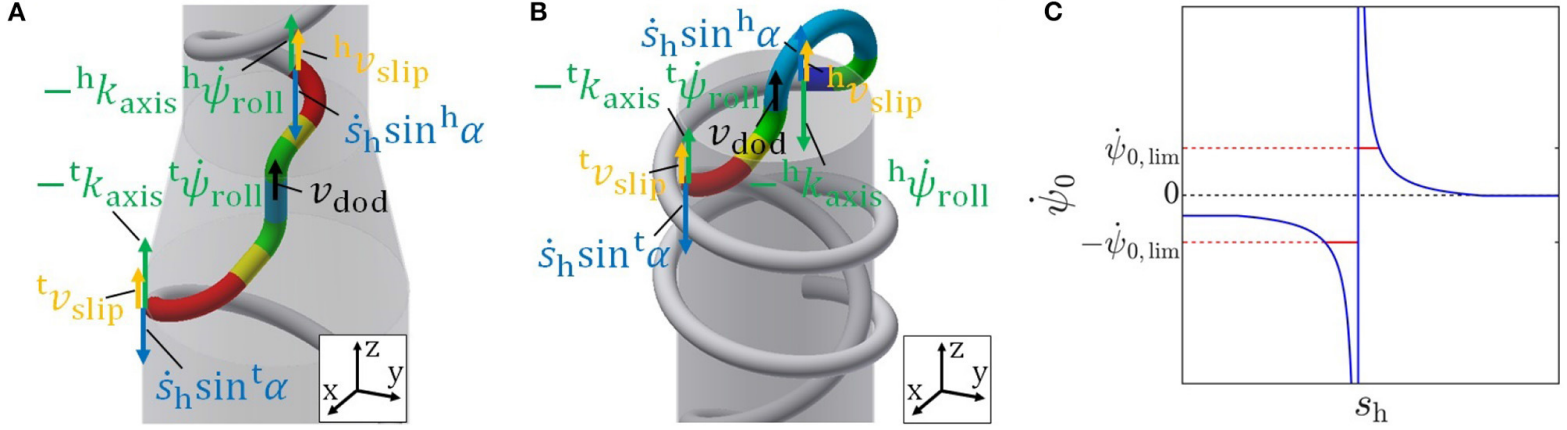
**(A)** Velocity of the dodging part in the axial direction in the case of a change in diameter. **(B)** Velocity of the dodging part in the axial direction in the case of inside-out motion. **(C)** ${\overset{∙}{\psi }}_{0}$ for the inside-out motion obtained by (42).

In the case of a change in diameter, the velocity of the dodging part in the axial direction, as shown in Figure 6A, is represented as

(27) vdod=−ktaxisψ˙troll−s˙hsintα+vtslip    =−khaxisψ˙hroll−s˙hsinhα+vhslip.

Using (23), (24), and (26), (27) is rewritten as

(28) vdod=−ktaxisψ˙0−(ktaxisτtw+sintα)s˙h+vtslip

(29)          =−khaxisψ˙0−(khaxisτhw+sinhα)s˙h−ltwlhwvtslip          −khaxisϕ˙diff.

Moreover, ${\overset{∙}{\phi }}_{\text{diff}}$ is expressed using ${\overset{∙}{\psi }}_{0}$, ṡ_h_, and ω_slip_ by substituting (20) into (25) as

(30) $$\begin{array}{l} {\overset{∙}{\phi }}_{\text{diff}}=A{\overset{∙}{\psi }}_{0}+B{\overset{∙}{s}}_{\text{h}}+\omega_{\text{slip}}, \end{array}$$

where

(31) A=−ktrot−khrot1−khrot,B=−ktrotτtw+2 costαdtpipe+khrotτhw−2 coshαdhpipe1−khrot.

Eventually, by substituting (30) into (29), (27) is expressed as

(32) vdod=−ktaxisψ˙0−(ktaxisτtw+sintα)s˙h+vtslip

(33)           =−khaxis(1+A)ψ˙0−{khaxis(τhw+B)+sinhα}s˙h          −ltwlhwvtslip−khaxisωslip.

As mentioned initially, our purpose is to derive ${\overset{∙}{\psi }}_{0}$ and ${\overset{∙}{\phi }}_{\text{diff}}$ as functions of the input value in Step 4 ṡ_h_, to realize the fixing of the dodging part, i.e., *v*_dod_ = 0. In addition to three equations, (30), (32), and (33), another equation is needed to designate four variables, ${\overset{∙}{\psi }}_{0}$, ${\overset{∙}{\phi }}_{\text{diff}}$, ^t^*v*_slip_, and ω_slip_. We introduce the control of the balance between the slip in the axial direction ^t^*v*_slip_ and slip around the axis ω_slip_ as another equation. There is a trade-off relationship between ^t^*v*_slip_ and ω_slip_, and cannot be zero at the same time in the case of the different radii of the head and tail winding parts. Here, we deal with two extreme instances: (A) a motion that no slip occurs around the axis but slip occurs in the axial direction (^t^*v*_slip_ ≠ 0, *ω*_slip_ = 0) and (B) a motion that where no slip occurs in the axial direction but slip occurs around the axis (^t^*v*_slip_ = 0, *ω*_slip_ ≠ 0). In (A), the slip in the axial direction ^t^*d*_pipe_ should be derived based on the slip model to fix the dodging part, whereas the slip around the axis ω_slip_ is determined to be zero. In (B), the slip around the axis ω_slip_ should be derived based on the slip model to fix the dodging part, whereas the slip in the axial direction ^t^*v*_slip_ is determined to be zero.

**(A) No slip around the axis but slip in the axial direction:**

We discuss the motion of no slip around the axis but slip in the axial direction (^t^*v*_slip_ ≠ 0, *ω*_slip_ = 0) first. Since the position of the dodging part in the axial direction is designed to be fixed relying on the slip in the axial direction ^t^*v*_slip_, the position is possible to be moved if there is the modeling error of the slip in the axial direction. On the other hand, the direction of the dodging part is able to be fixed precisely because there is no slip around the axis. Therefore, this motion is suitable for navigating around an obstacle for which the direction of the dodging part should be neatly fixed. In this motion, from (32), (33), and ω_slip_ = 0, the velocity of the dodging part is represented as

(34) vdod=−Ftltw−Fhlhwltw+lhwψ˙0−Gtltw−Ghlhw(1−khrot)(ltw+lhw)s˙h.

where

(35) Ft=ktaxis,Fh=1−ktrot1−khrotkhaxis,Gt=ktaxisτtw+sintα,Gh=khaxis1−khrot(−ktrotτtw+2 costαdtpipe+hτw−2 coshαdhpipe)     +sinhα.

Therefore, ${\overset{∙}{\psi }}_{0}$ to fix the dodging part in the axial direction, i.e., to realize *v*_dod_ = 0, is obtained by

(36) ψ˙0=−Gtltw+GhlhwFtltw+Fhlhws˙h.

Then, ${\overset{∙}{\phi }}_{\text{diff}}$ is calculated by substituting ω_slip_ = 0 and (36) into (30) as

(37) ϕ˙diff=Aψ˙0+Bs˙h, =(−Gtltw+GhlhwFtltw+FhlhwA+B)s˙h.

**(B) No slip in the axial direction but slip around the axis:**

Next, let us consider the motion of no slip in the axial direction but slip around the axis (^t^*v*_slip_ = 0, *ω*_slip_ ≠ 0). Since the direction of the dodging part is designed to be fixed relying on the slip around the axis ω_slip_, the direction is possible to be moved if there is the modeling error of the slip around the axis. On the other hand, the position of the dodging part in the axial direction is able to be fixed precisely because there is no slip in the axial direction. Hence, this motion is effective when the position of the dodging part in the axial direction has to be maintained primarily, e.g., a change in diameter whose target form is axially symmetric as described later. Using (28), (29), and ^t^*v*_slip_ = 0, ${\overset{∙}{\psi }}_{0}$ and ω_slip_ to fix the dodging part are obtained by

(38) ψ˙0=−(τtw+sintαktaxis)s˙h,

(39) ϕ˙diff=(τtw+sintαktaxis−τhw−sinhαkhaxis)s˙h.

Note that these two motions (A) and (B) are equal to each other in the junction, bend, shear, and blockage, which can be negotiated without any slip in the axial direction and around the axis. For these cases, ^t^*v*_slip_ and ${\overset{∙}{\phi }}_{\text{diff}}$ become zero, and the same result is derived as in section 4.2.1.

##### 4.2.3.2. Motion Between Inside and Outside

We next consider a case of inside-out motion. As in the case of a change in diameter, the equilibrium of force in the axial direction is described as (26). Then, the velocity of the two ends of the dodging part, as shown in Figure 6B, is represented as

(40) vdod=−ktaxisψ˙troll−s˙hsintα+vtslip=khaxisψ˙hroll+s˙hsinhα+vhslip.

**(A) No slip around the axis but slip in the axial direction:**

We begin, as before, with the motion of no slip around the axis but slip in the axial direction (^t^*v*_slip_ ≠ 0, *ω*_slip_ = 0). From (26), (40), and ω_slip_ = 0, the velocity of the dodging part is represented as

(41) vdod=−Ftltw−Fhlhwltw+lhwψ˙0−Gtltw−Ghlhwltw+lhws˙h.

Therefore, ${\overset{∙}{\psi }}_{0}$ and ${\overset{∙}{\phi }}_{\text{diff}}$ to realize *v*_dod_ = 0 is derived by

(42) ψ˙0=−Gtltw−GhlhwFtltw−Fhlhws˙h,

(43) ϕ˙diff=(−Gtltw−GhlhwFtltw−FhlhwA+B)s˙h.

Here, ${\overset{∙}{\psi }}_{0}$ ends up diverging when the denominator ^t^*F*^t^*l*_w_ − ^h^*F*^h^*l*_w_ becomes zero, as shown in Figure 6C. Therefore, we introduce the limitations $-{\overset{∙}{\psi }}_{0,\text{lim}}$ and ${\overset{∙}{\psi }}_{0,\text{lim}}$ for ${\overset{∙}{\psi }}_{0}$ so as not to require a rapid change in the joint angle that the actuator cannot realize. The effect of this limitation must be considered. Since *v*_dod_ is a linear function of ${\overset{∙}{\psi }}_{0}$, the sign of *v*_dod_ is determined by the coefficient of ${\overset{∙}{\psi }}_{0}$ and whether ${\overset{∙}{\psi }}_{0}$ is larger or smaller than ${\overset{∙}{\psi }}_{0}$ to realize *v*_dod_ = 0 (42). Before the divergence, ^t^*F*^t^*l*_w_ − ^h^*F*^h^*l*_w_ is positive and the coefficient of ${\overset{∙}{\psi }}_{0}$ in (41) becomes positive. Here, ^t^*l*_w_ + ^h^*l*_w_ > 0 satisfies because ^t^*l*_w_ and ^h^*l*_w_ are the lengths of the winding parts within the approximation range of the robot. $-{\overset{∙}{\psi }}_{0,\text{lim}}$ is smaller than ${\overset{∙}{\psi }}_{0}$ obtained by (42), as shown in Figure 6C. After the divergence, ^t^*F*^t^*l*_w_ − ^h^*F*^h^*l*_w_ is negative, the coefficient of ${\overset{∙}{\psi }}_{0}$ in (41) becomes negative. ${\overset{∙}{\psi }}_{0,\text{lim}}$ is larger than ${\overset{∙}{\psi }}_{0}$ obtained by (42), as shown in Figure 6C. Therefore, *v*_dod_ becomes negative while the limitation is imposed on ${\overset{∙}{\psi }}_{0}$. This *v*_dod_ < 0 indicates that both the head and tail winding parts move to the side of the pipe until segment-5 contacts the pipe's edge. This phenomenon only fixes the dodging part and doesn't interfere with overall motions, such as the robot falling out of the pipe.

**(B) No slip in the axial direction but slip around the axis:**

We next consider the motion of no slip in the axial direction but slip around the axis (^t^*v*_slip_ = 0, *ω*_slip_ ≠ 0). This motion is also suitable for the inside-out motion because its target form is axially symmetric, as described later, and the displacement of the dodging part in the axial direction has to be maintained more appropriately than the rotation of the dodging part around the axis. Using (40), ${\overset{∙}{\psi }}_{0}$ and ${\overset{∙}{\phi }}_{\text{diff}}$ to fix the dodging part, i.e., to realize *v*_dod_ = 0, are obtained by (38) and (39), the same equations as in the case of the change in diameter.

Note that the position in the axial direction of the dodging part can be compensated by the rolling motion, and the direction of the dodging part can be compensated by ϕ_offset_ if the dodging part deviates from the appropriate position and direction during movement.

## 5. Form Design for Application

In this section, we present the design of the dodging parts for a junction, bend, shear, blockage, and change in pipe diameter and the design of a guiding part for outside the pipe for the motion between the inside and outside of a pipe as examples of applications of the proposed motion.

### 5.1. Junction and Bend

**Figure 7:**
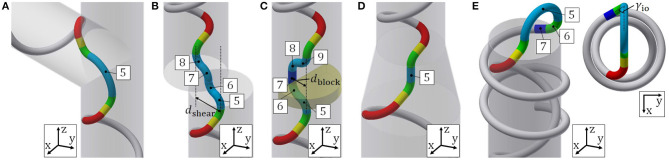
Segment configuration for **(A)** junction and bend, **(B)** shear, **(C)** blockage, **(D)** change in diameter, and **(E)** inside-out motion.

The target form for a junction and bend is presented in Figure 7A. The dodging part is composed of segment-5, an arc segment, whose parameters are (*r*_*j*_, ϕ_*j*_) = (*r*_bend_, ϕ_bend_) and ${\hat{\psi }}_{5}=\pi {-}^{\text{h}}\phi_{\text{rot}}$. For a junction, *r*_bend_ is the outer radius of a pipe and ϕ_bend_ is the bending angle of the junction. For a bend, *r*_bend_ is the radius and ϕ_bend_ is the bending angle of the bend.

### 5.2. Shear

The target form for shear is shown in Figure 7B. The dodging part is composed of segments-5–8, which are arc segments whose parameters are (*r*_*j*_, ϕ_*j*_) = (*r*_c_, γ_s_), ${\hat{\psi }}_{5}={\hat{\psi }}_{6}={\hat{\psi }}_{7}=\pi$, and ${\hat{\psi }}_{8}=\pi {-}^{\text{h}}\phi_{\text{rot}}$. The dodging part is defined by the distance between the two axes of pipes *d*_shear_. γ_s_ is calculated from,

(44) $$\begin{array}{l} \gamma_{\text{s}}=\arccos\left(1-\frac{d_{\text{shear}}}{4r_{\text{c}}}\right). \end{array}$$

### 5.3. Blockage

The target form for a blockage is illustrated in Figure 7C. The dodging part is composed of four arc segments-5, 6, 8, 9, whose parameters are (*r*_*j*_, ϕ_*j*_) = (*r*_c_, γ_block_) and (${\hat{\psi }}_{5},{\hat{\psi }}_{6},{\hat{\psi }}_{8},{\hat{\psi }}_{9}$) = ($\pi ,0,0,\pi ,-\frac{\pi }{2}{-}^{\text{h}}\phi_{\text{rot}}$), and a straight line segment-7, whose parameters are *l*_*j*_ = *l*_block_ and ${\hat{\psi }}_{7}=0$. The dodging part is determined by its width *d*_block_, which is defined as the length between the axis of the pipe and the straight line segment parallel to the axis, and *l*_7_ = *l*_block_, depending on the shape of the blockage. The geometric parameter γ_block_ is calculated from,

(45) $$\begin{array}{l} \gamma_{\text{block}}=\arccos\left(1-\frac{d_{\text{block}}}{2r_{\text{c}}}\right). \end{array}$$

### 5.4. Change in Pipe Diameter

The target form for a change in pipe diameter is shown in Figure 7D. The dodging part is composed of segment-5, a straight line segment, whose parameters are *l*_5_ = *l*_dc_ and ${\hat{\psi }}_{5}=0$. Since the dodging part is the straight line segment and ${\hat{\psi }}_{5}=0$, ${\hat{\psi }}_{4}$ is determined by ${\hat{\psi }}_{4}=\pi +\phi_{\text{offset}}+\phi_{\text{diff}}$ instead, where ϕ_diff_ is the time integral of ${\overset{∙}{\phi }}_{\text{diff}}$. The determination of the dodging part depends on the length of the part of the pipe where the diameter changes. This form is axisymmetric, and the direction of the dodging part does not matter. Hence we set ϕ_offset_ = 0. Eventually, ${\hat{\psi }}_{4}$ is determined by ${\hat{\psi }}_{4}=\pi +\phi_{\text{diff}}$.

### 5.5. Motion Between Inside and Outside

Expanding on the proposed motion, we propose a motion that corresponds to passing between the inside and outside of a pipe. This motion is useful when a pipe opening is difficult to approach directly or when a pipe needs to be inspected from both inside and outside. The target form for this motion is illustrated in Figure 7E, and the parameters for each segment are shown in Table 2. To begin, we design the guiding part for outside the pipe. Here segments-5–7 comprise the head guiding part, which is fixed by the radius of segment-5 *r*_io_. γ_io_ and *l*_io_ are given by

(46) γio=arcsinrhw2rio,

(47) $$\begin{array}{l} l_{\text{io}}=\frac{\sqrt{{\left(2r_{\text{io}}\right)}^{2}{-}^{\text{h}}r_{\text{w}}^{2}}-r_{\text{c}}\left\{1-\cos\left(\frac{\pi }{2}{-}^{\text{h}}\alpha \right)\right\}}{\cos^{\text{h}}\alpha }. \end{array}$$

Here, *r*_io_ should be determined so that *l*_io_ ≥ 0 holds. In this way, the proposed motion available for an arbitrary dodging part can be expanded to outside the pipe by designing guiding part for the outside. This target form does not have a dodging part, and the tail guiding part is directly connected to the head guiding part. Therefore, this motion can also be realized in the same way as for inside the pipe, by determining ${\hat{\psi }}_{4}=\pi +\phi_{\text{diff}}$. For the motion from outside to inside a pipe, the tail guiding parts are composed of segments listed in the opposite order in Table 2.

**Table 2:** Parameters of the segments comprising the head guiding part for the outside of a pipe.

**Seg. no. j**	**Type**	**Parameter**	**${\hat{\psi }}_{j}$**
5	Circular arc	(*r*_*j*_, ϕ_*j*_) = (*r*_io_, π)	γ_io_
6	Circular arc	$\left(r_{j},\phi_{j}\right)=\left(r_{\text{c}},\frac{\pi }{2}{-}^{\text{h}}\alpha \right)$	0
7	Straight line	*l*_*j*_ = *l*_io_	$-\frac{\pi }{2}$

## 6. Experimental Results

**Figure 8:**
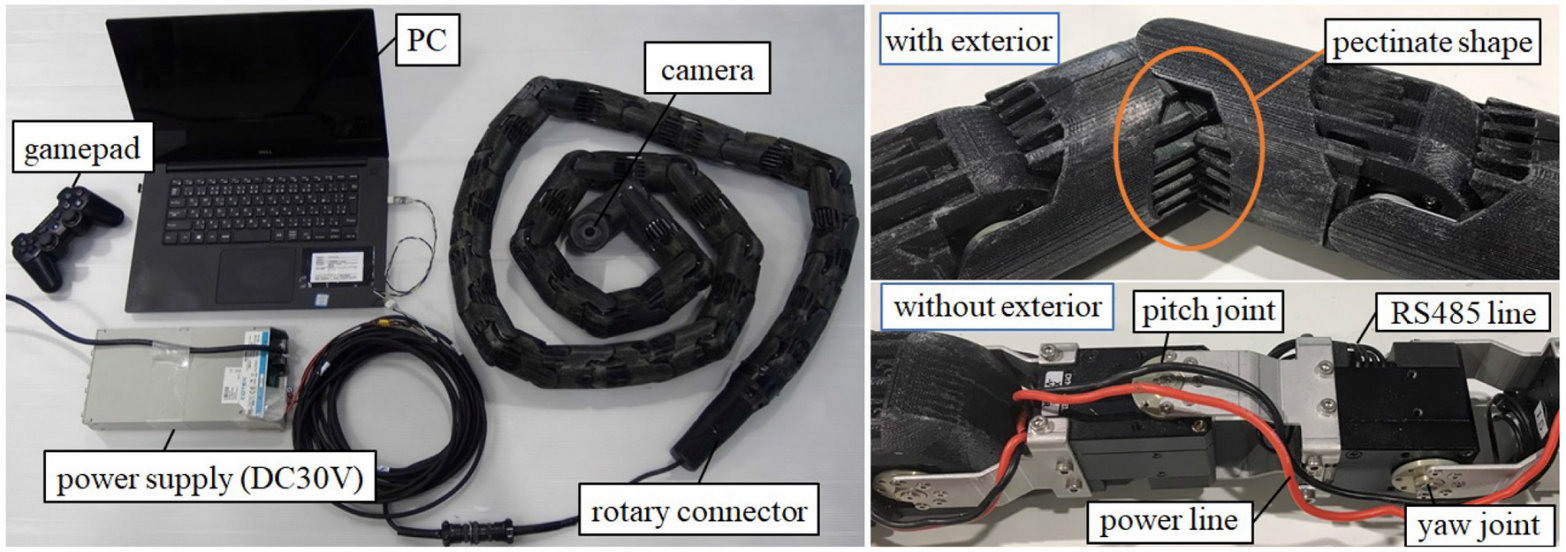
System configuration of a snake robot developed in Takemori et al. (2018b).

We performed experiments to verify the effectiveness of the proposed method. The system configuration of a snake robot is illustrated in Figure 8. We used the snake robot developed in Takemori et al. (2018b). The snake robot has a module configuration, which has a joint and link covered by an exterior. This exterior has a pectinate shape, providing a smooth surface without affecting the bending of the joint. The number of joints is 36, the link length is 70 mm, the diameter of the link is 56 mm, the weight per link is 150 g, the maximum torque of a joint is 4.0 Nm, and the maximum bending angle of a joint is 90°. The motor was driven by the position control with the limitation of the current (0.3 A) to allow the compliance of the joint, and the PID gains are set as (P, I, D) = (800, 0, 100). The snake robot is powered via a cable, and the target angle for each joint is sent from a computer via an RS485 interface. The camera is mounted on the head to inspect the pipe and to help the operator control the robot remotely. The operator uses a gamepad to perform an operation.

The pipes used most have an inner diameter of 194 mm and an outer diameter of 200 mm. The only pipe used in the experiments involving a change in pipe diameter had an inner diameter of 290 mm. The parameters used in the experiments are listed in Table 3. Here, index m means t or h. ^m^*k*_axis_ and ^m^*k*_rot_ for each pipe were measured in the preliminary experiments in which only the rolling motion was performed. We determined *r*_c_ = 90 mm for all subsequent cases.

**Table 3:** Parameters used in experiments.

**Pipe diameter**	**^m^*r*_w_** **mm**	**^m^*p*_w_** **mm**	**^m^*r*_in_** **mm**	**^m^*k*_axis_** **mm/rad**	**^m^*k*_rot_** **–**
194 mm (inside)	69	501	31	24.2	0.348
194 mm (inside)	69	600	31	17.2	0.343
200 mm (outside)	128	150	–	37.8	0.0111
290 mm (inside)	117	556	53	27.1	0.187

We measured the static coefficient between the robot surface and inner wall of the pipe. We put one unit of the robot, which is composed of one link and one joint, on the pipe and measured the tilted angle of the pipe when the unit started sliding. The unit started sliding when the tilted angle was about 16°, and then, the measured static coefficient was arctan16° = 0.27. This value is the reference value because it seems to be easily changed according to the condition of contact.

### 6.1. Evaluation of Proposed Slip Model

**Figure 9:**
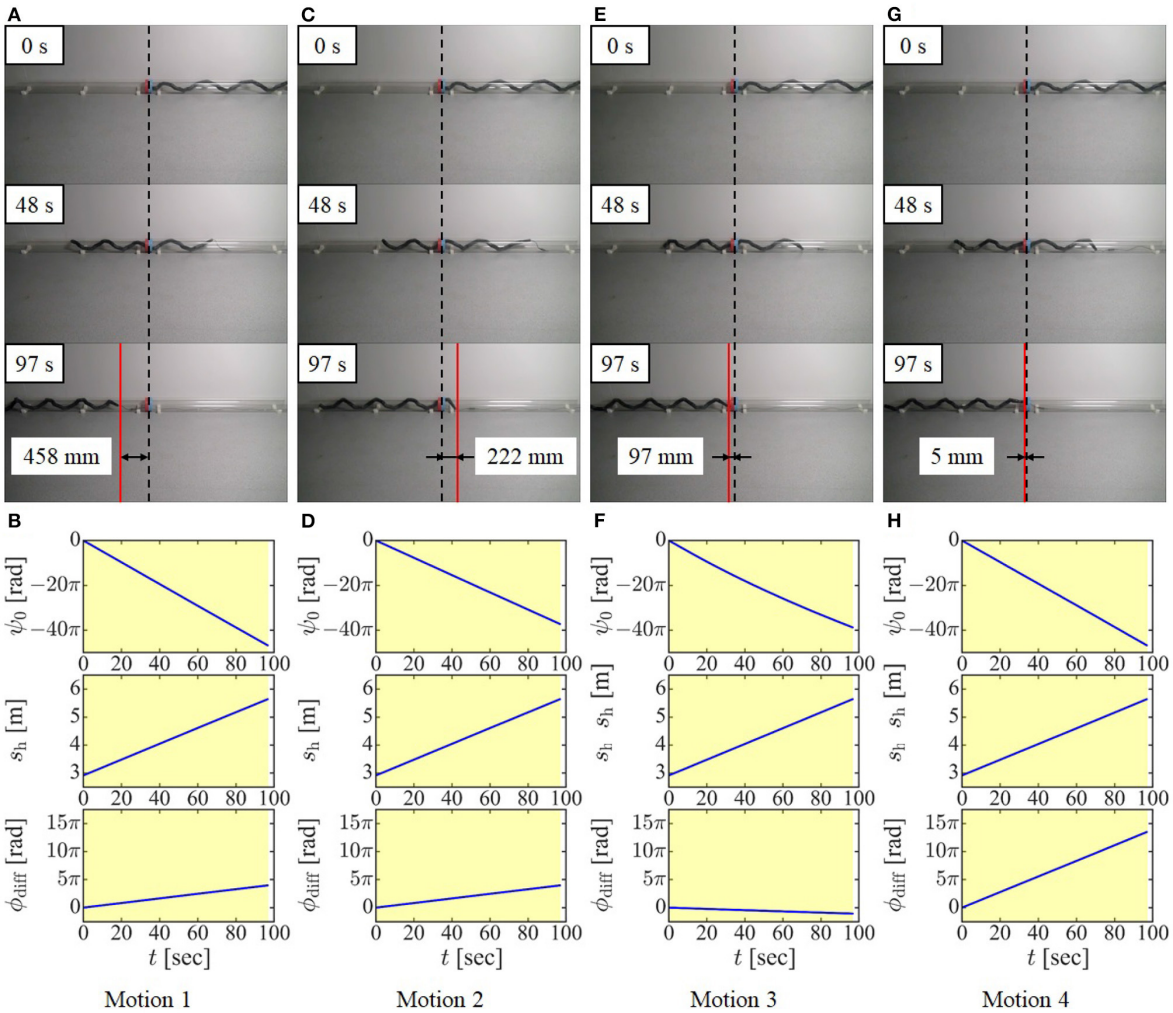
Experimental results and data from preliminary experiments. **(A,B)** Motion 1: fixing the dodging part only on the tail winding part, **(C,D)** Motion 2: fixing the dodging part only on the head winding part, **(E,F)** Motion 3: employing the proposed slip model (^t^*v*_slip_ ≠ 0, *ω*_slip_ = 0), and **(G,H)** Motion 4: employing the proposed slip model (^t^*v*_slip_ = 0, *ω*_slip_ ≠ 0).

First, we conducted experiments to verify the model considering the slip in cases where the head and tail winding parts have different radii based on the displacement of the dodging part in the axial direction. If two pipes have different radii, it is physically impossible for a winding part having a larger radius to enter the smaller pipe. In this case, the displacement of the dodging part toward the smaller pipe is altered constrainedly and cannot be observed correctly. Therefore, two pipes having the same diameter, 194 mm, were used instead, and the tail winding part, for this experiment only, had a larger pitch (^t^*p*_w_ = 600 mm) than the head winding part (^h^*p*_w_ = 501 mm). To clearly show the displacement of the dodging part, the head of the robot was located between the ends of two pipes at the beginning of the experiment, as indicated by the dotted line in Figure 7. Then, the shift control, combined with the rolling motion and the change in ${\hat{\psi }}_{4}$ calculated in Motions 1–4 [(Motion 1) considering the displacement only on the tail winding part (23), (Motion 2) considering the displacement only on the head winding part (24), (Motion 3) considering the displacement with the slip between the robot and pipe in the motion of no slip around the axis but slip in the axial direction (^t^*v*_slip_ ≠ 0, *ω*_slip_ = 0), and (Motion 4) considering the displacement with the slip in the motion of no slip in the axial direction but slip around the axis (^t^*v*_slip_ = 0, *ω*_slip_ ≠ 0)], was conducted in Step 4 until the tail of the robot reached the head guiding part. Considering the target form of the robot as shown in Figure 7D when *l*_dc_ = 0 mm, the tail of the robot is located just between the ends of two pipes at the end of the experiment if the dodging part is fixed properly. Therefore, we measured the position of the tail of the robot at the end of each experiment, as indicated by the red line in the image, and compared it under four conditions. Note that this experiment is focused on the fixing of the position of the dodging part only in the axial direction since it is difficult to observe the change of the direction of the dodging part in the axially symmetric target form. The results and data of these experiments are shown in Figure 9. When the displacement of the dodging part was considered on either the tail winding part or the head winding part (Motion 1 and Motion 2), the error was 458 or 222 mm, respectively. On the other hand, the proposed model (^t^*v*_slip_ ≠ 0, *ω*_slip_ = 0) (Motion 3) produced less error, 97 mm. Furthermore, the proposed model considering the slip (^t^*v*_slip_ = 0, *ω*_slip_ ≠ 0) (Motion 4) produced the smallest error, 5 mm. These results indicated that Motion 1 and Motion 2 produces the large errors because they ignore the effect of the slip. Motion 3 reduced the error compared with the first two motions by fixing the dodging part in consideration of the slip. However, a small error was left due to the modeling error caused by the viscous friction model or the condition of the contact in the axial direction because Motion 3 is relying on the slip in the axial direction. In contrast, Motion 4 successfully realized the fixing of the dodging part in the axial direction as expected because it did not require the slip in the axial direction and was not affected by the modeling error of the slip in the axial direction. Consequently, the proposed motions based on the slip model both in the cases of (^t^*v*_slip_ ≠ 0, *ω*_slip_ = 0) and (^t^*v*_slip_ = 0, *ω*_slip_ ≠ 0) are regarded as effective for fixing the dodging part. The reduction of the modeling error of the slip is left as our future task.

### 6.2. Experiments for Various Pipe Structures

We then performed four experiments in which the robot negotiated a junction, a shear, a blockage, and a discontinuous change in diameter. As mentioned in section 5, the target form for a bend is similar to that for a junction, and the target form for a continuous change in pipe diameter is similar to that for a discontinuous change in pipe diameter. Therefore, these four experiments can demonstrate the effectiveness of the proposed method for all pipe structures in Figure 1A. Also, we performed an experiment in which the movement is from inside to outside a pipe. The operator looked at the snake robot directly and performed the operation according to the procedure described in the first part of section 4.2.

**Figure 10:**
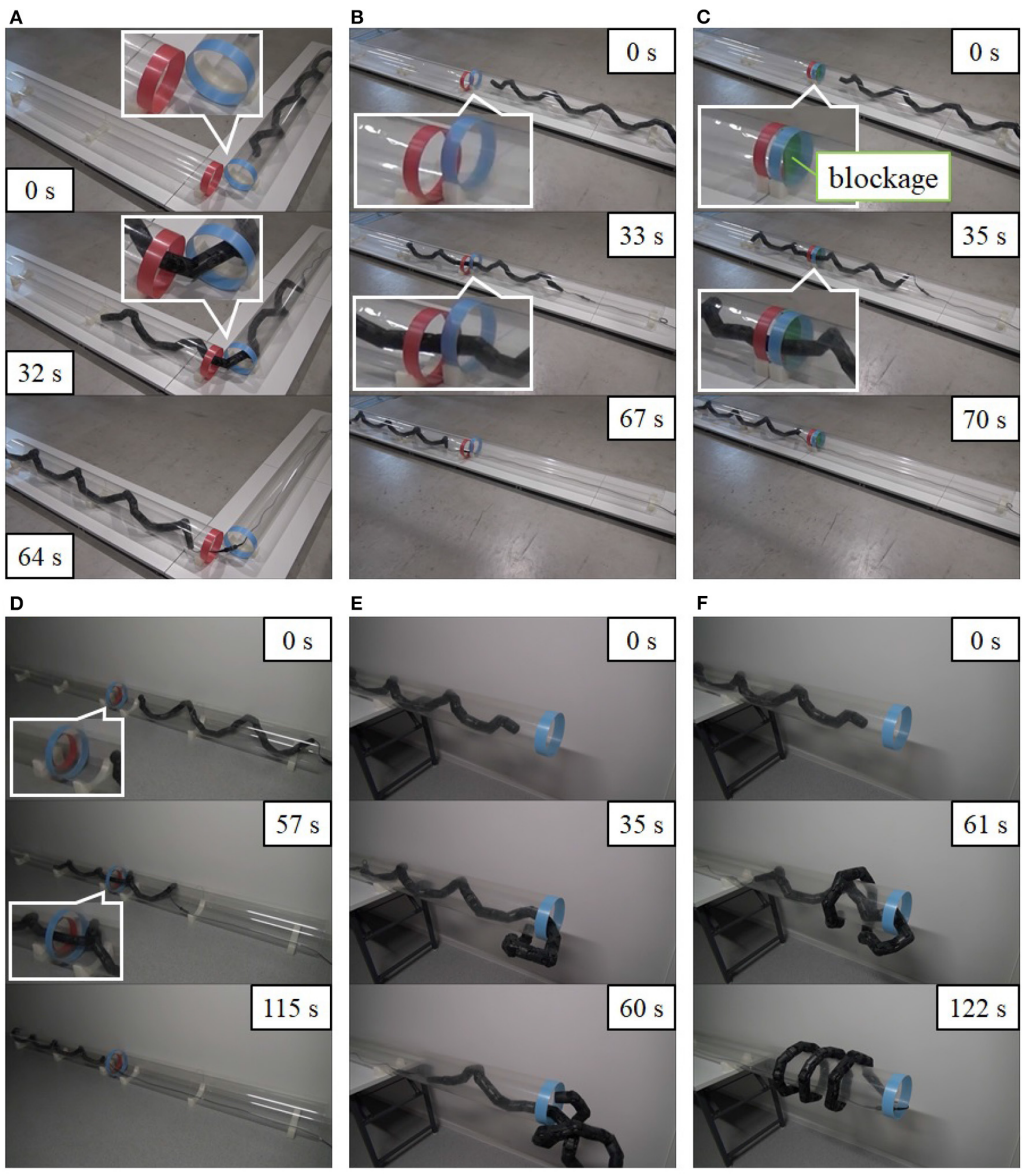
Experimental results of **(A)** negotiating a junction (*r*_bend_ = 100 mm and $r_{\text{bend}}=90^{∙}$), **(B)** negotiating shear (*d*_shear_ = 100 mm), **(C)** negotiating a blockage (the right half of the pipe is blocked in width by 10 mm, and *d*_block_ = 48.5 mm, *l*_block_ = 30 mm), **(D)** negotiating a change in diameter (pipe inner diameter changes from 290 to 194 mm, and *l*_dc_ = 0 mm) using the proposed slip model (^t^*v*_slip_ ≠ 0, *ω*_slip_ = 0), **(E)** moving from inside to outside a pipe using the proposed slip model (^t^*V*_slip_ ≠ 0, ω_slip_ = 0, *r*_io_ = 90 mm, ${\overset{∙}{\psi }}_{0,\text{limit}}=0.03$ rad/s), and **(F)** moving from inside to outside a pipe using the proposed slip model (^t^*V*_slip_ ≠ 0, ω_slip_ = 0, *r*_io_ = 90 mm).

**Figure 11:**
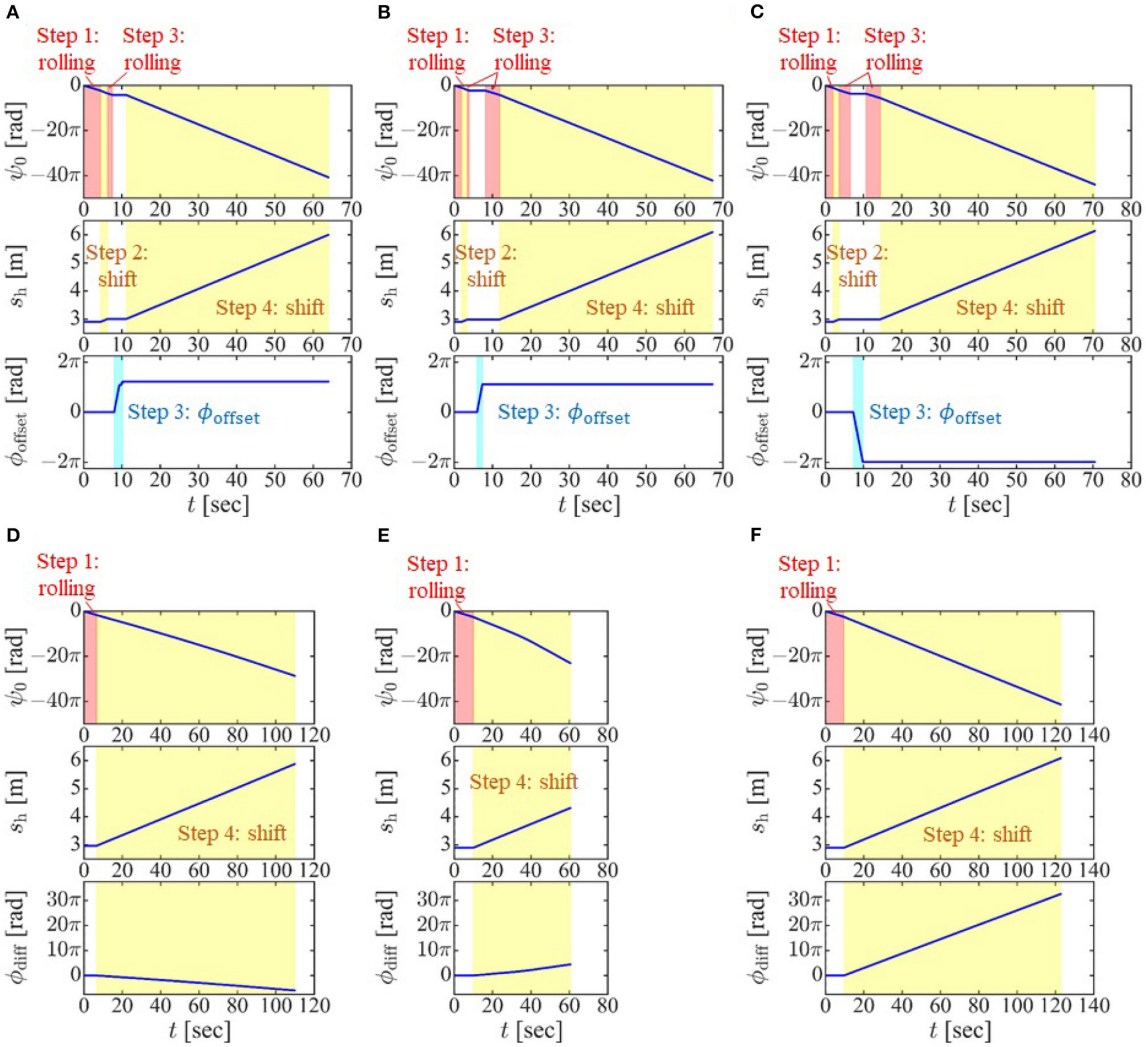
Experimental data on **(A)** negotiating a junction, **(B)** negotiating a shear, **(C)** negotiating a blockage, **(D)** negotiating a change in diameter using the proposed slip model (^t^*v*_slip_ ≠ 0, *ω*_slip_ = 0), **(E)** moving from inside to outside a pipe using the proposed slip model (^t^*v*_slip_ ≠ 0, *ω*_slip_ = 0), and **(F)** moving from inside to outside a pipe using the proposed slip model (^t^*V*_slip_ = 0, *ω*_slip_ ≠ 0).

As shown in Figures 10A–D, the snake robot successfully negotiated the junction, shear, blockage, and change in pipe diameter. The snake robot was also able to move from the inside to the outside of the pipe, as shown in Figure 10F. Please also see the Supplementary Video 1 for details. Figure 11 indicates the values of ψ_0_, *s*_h_, and ϕ_offset_ for each experiment. For the experiments involving a junction, shear, and blockage, the robot was able to negotiate the pipe under shift control and rolling motion, as described in section 4.2, with only the first adjustment by the operator of the position and direction of the dodging part in Step 3.

**Figure 12:**
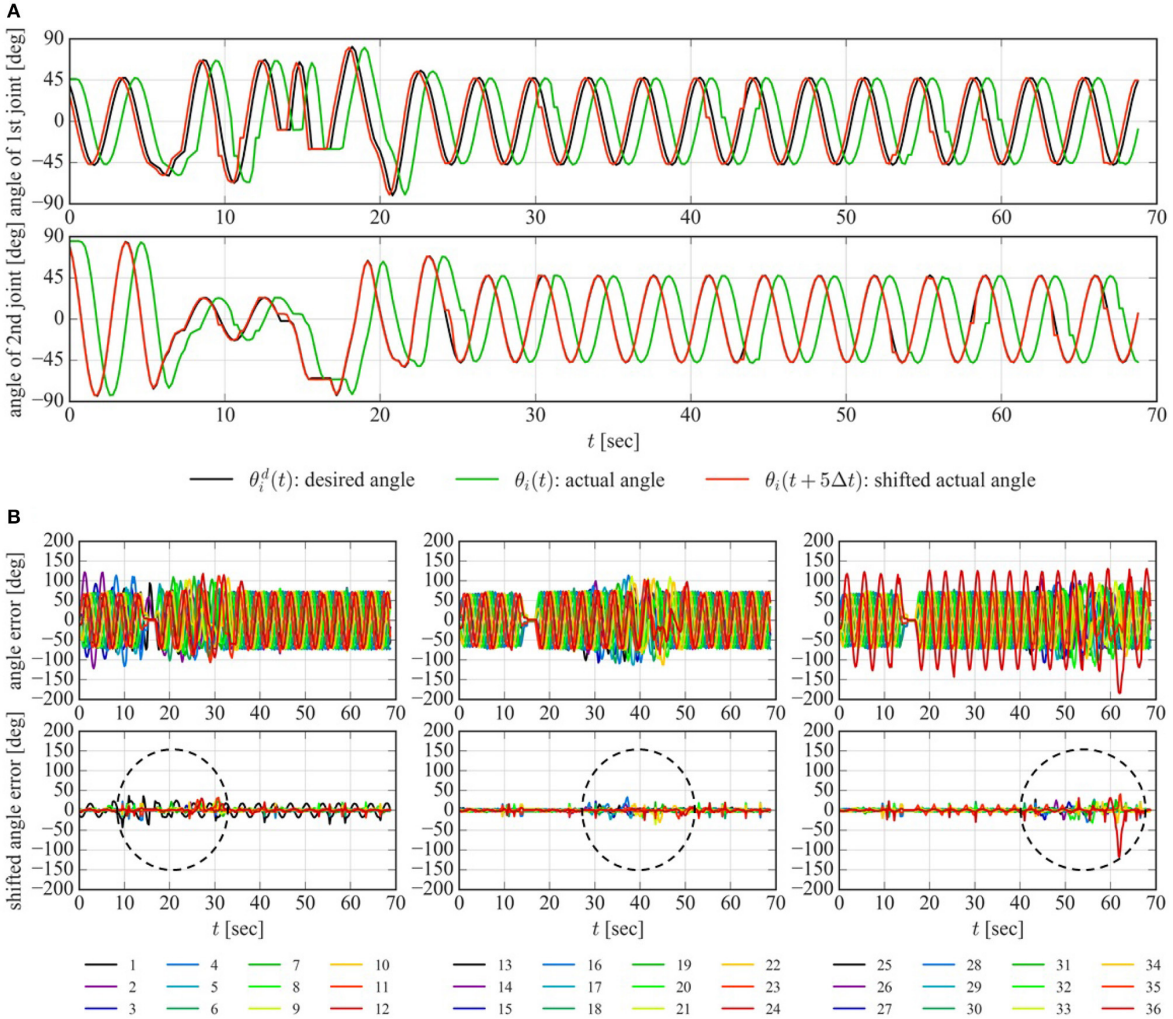
Experimental data of the joint angle for negotiating a junction. **(A)** Time delay of the joint angle for the first joint (top) and the second joint (bottom). **(B)** Angle error of the joint between the desired angle and the actual angle of the joint (top). and shifted angle error of the joint between the desired angle and the actual angle moved forward 5 time steps (bottom). Angle error and shifted angle error of the 1–12th joints, the 13–24th joints, and the 25–36th joints are shown in the left, center, and right panel of the figures, respectively.

Since the junction is the severest obstacle that does not allow the slightest deviation of the position and direction of the dodging part, the experiment to verify the angle error of the joint between the desired angle and the actual angle was also conducted for negotiating a junction. Due to the limitation of the communication speed, the time step of this experiment (Δ*t* = 0.2 s) is 10 times as large as that of the other experiments to obtain the joint angle. As shown in Figure 12B, the actual angle of the joint θ_*i*_(*t*) (green line) lagged behind the desired angle $\theta_{i}^{d}\left(t\right)$ (black line). This steady delay is thought to be caused by the communication delay to send the desired angle to each joint and receive the actual angle from each joint and the time delay needed to change the angle of each joint from the actual angle to the desired angle due to the limitation of the speed of the motor. The length of the time delay was about five steps for every joint except the first joint, which responded one time step earlier than the others. The actual angle moved forward five steps θ_*i*_(*t* + 5Δ*t*) (red line) matched the desired angle well for the second joint as shown in the bottom of the Figure 12A, although the error about one time step was left only for the first joint. The angle error between the desired and actual angle for each joint ($\theta_{i}\left(t\right)-\theta_{i}^{d}\left(t\right)$) is depicted in the top of the Figure 12B. To eliminate the effect of the time delay, the angle error between the desired and shifted actual angle for five time steps for each joint ($\theta_{i}\left(t\right)-\theta_{i}^{d}\left(t+5\Delta t\right)$) is depicted in the bottom of the Figure 12B. As shown in the bottom of the Figure 12B, the comparatively large angle error of the joint was moved from head to tail with the passage of time. This indicates that the angle error was observed near the dodging part as enclosed with dotted lines in Figure 12B and the dodging part deviates from the appropriate position and direction to some extent. In addition, the error of the last joint, 36th joint, is quite large at *t* = 62 s. This is because the tail link is longer than the other links and was caught by the pipe when it passed through the junction. The error of the position and direction of the dodging part is thought to be compensated by two factors caused by the robot's geometric constraints: the compliant adaptation to the environment at the joint thanks to the position control of the motor with the limitation of the torque, and the slippage between the robot and the pipe.

Also, the robot was able to negotiate the change in diameter with the proposed model considering the slip between the robot and the pipe even when the radii of the head and tail winding parts differed. Figure 10D shows only the result of the proposed model (^t^*v*_slip_ ≠ 0, *ω*_slip_ = 0), but the model (^t^*v*_slip_ = 0, *ω*_slip_ ≠ 0) also worked successfully; see Supplementary Video 1. In contrast, the robot failed to move from inside to outside the pipe using the proposed slip model (^t^*v*_slip_ ≠ 0, *ω*_slip_ = 0), as in Figure 10E, and succeeded to do so only with the proposed slip model (^t^*v*_slip_ = 0, *ω*_slip_ ≠ 0) Figure 10F. As shown in the middle panel of Figure 10E, the robot was unable to support the part of itself that was below the pipe due to the torque limitation of the motor, and the head winding part did not contact the pipe and receive the friction force, contrary to what we had expected. Hence, the dodging part moved the right side of the image, although in theory it was expected to move to the left side of the image after ${\overset{∙}{\psi }}_{0}$ reached $-{\overset{∙}{\psi }}_{0,\text{limit}}$. This effect led the robot to fall out of the pipe. On the other hand, since the experiment shown in Figure 10F was conducted using the proposed slip model (^t^*v*_slip_ = 0, *ω*_slip_ ≠ 0), the dodging part did not deviate from the proper position in the axial direction even though the robot was not always able to press its body against the pipe. Instead, the rotation of the robot around the axis was found to be caused by the slip around the axis. In addition, due to the torque limitation of the motor, the robot is likely to failed to support the head winding part for both Figures 10E,F of the revised paper depending on the initial orientation of the robot and the protective function of the motor, which made the motor output torque zero when the motor detects the persistent load that exceeds maximum output.

## 7. Conclusion

A unified approach was proposed for designing the motion that enables a snake robot to negotiate complicated pipe structures. The proposed method enables the robot to overcome various obstacles by designing the dodging part, which is part of the target form, specifically for the obstacle. To realize this, both ends of the dodging part are arranged on the axes of the pipes with guiding parts. In addition, we developed a method of fixing the dodging part to an obstacle during obstacle negotiation that involved an appropriate combination of rolling motion and shift control. Also, we constructed a model considering slippage between robot and pipe, and expanded the proposed method to make it applicable to motions that require two helices having different radii, i.e., the motion for change in diameter and the motion between inside and outside of a pipe. We conducted experiments to verify the effectiveness of these methods and demonstrated that the snake robot successfully negotiated not only a junction, which was already realized, but also a shear, a blockage, and a discontinuous change in pipe diameter, which were impossible previously. We also realized movement from inside to outside a pipe in an experiment.

We shall in a future study consider a way to conduct remote operations more easily. Currently, the operator has to adjust appropriately the relative position of the dodging part to the environment. Also, experiments are now conducted in the ideal situation where the operator can recognize the state of the robot by directly looking at the robot through the transparent pipe. We also leave as a future task the realization of autonomous movement by detecting a pipe structure using sensors given no parameter values. Finally, another task for the future is a kinematic/dynamic analysis of the motion to keep the appropriate contact with the pipe.

## Data Availability Statement

The original contributions presented in the study are included in the article/Supplementary Material, further inquiries can be directed to the corresponding author/s.

## Author Contributions

MI: writing the paper, developing the theory, and running experiments. TT: developing the theory, running experiments, providing feedback to theoretical and experimental results, and providing feedback on drafts of the report. MT and FM: developing the theory, providing feedback to theoretical and experimental results, and providing feedback on drafts of the report. All authors contributed to the article and approved the submitted version.

## Conflict of Interest

The authors declare that the research was conducted in the absence of any commercial or financial relationships that could be construed as a potential conflict of interest.

## Supplementary Material

Supplementary Video 1 Pipe negotiation.
